# Hardship Strengthens Mutual Bonds

**DOI:** 10.1371/journal.pbio.1001706

**Published:** 2013-11-05

**Authors:** Robin Meadows

**Affiliations:** 1 Freelance Science Writer, Fairfield, California, United States of America

Tiny sap-sucking insects that are a scourge to gardeners also have the upside of helping trees survive in seasonally dry forests in Central America. How? Scale insects use carbon they get from *Cordia alliodora* trees to make sugar-rich “honeydew” for *Azteca pittieri* ants, which in turn defend the trees against leaf-munching insects. Mutualism is often stronger when resources are scarce, but this interdependence usually involves a commodity that is traded directly between species. Now, in this issue of *PLOS Biology*, Pringle and colleagues show that lack of a resource that is not traded—water—intensifies the bonds between *C. alliodora*, scale insects, and ants.

**Figure:**
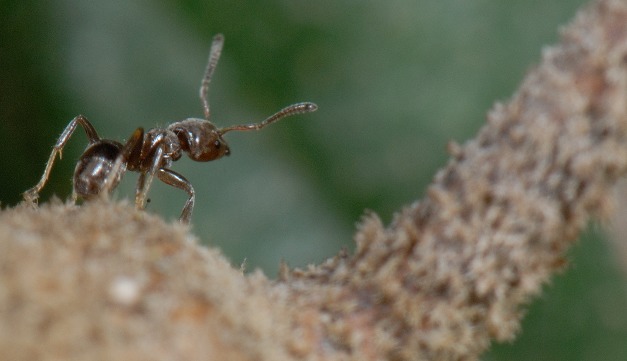
An *A. pittieri* ant patrols the surface of a *C. alliodora* tree stem in Mexico. Pringle et al. show that the strength of the defensive mutualism between *A. pittieri* ants and *C. alliodora* trees increases with water stress across Mesoamerica. *Image credit: Enrique Ramírez-García*.

Found from southern Mexico through South America, *C. alliodora* has stem hollows where ants nest and tend flocks of scale insects. Named for their protective coverings, scale insects are vampires to the vegetable kingdom, piercing plants with tubular mouths to drink straight from the vascular system. As they imbibe, they secrete honeydew for ants to harvest and eat. Rounding out this mutualistic circle, the ants patrol their *C. alliodora* host for beetle larvae, caterpillars, and other herbivores, biting them until they leave.

Previous studies suggested that plants may invest more carbon in ant defense during water stress. This scenario is particularly taxing for *C. alliodora*, which drops its leaves during the dry season and must make its carbon stores last long enough to grow new leaves during the next rainy season. But ant colonies must be maintained year-round to ensure defense of leaves during the growing season, safeguarding the production of carbon to get the trees through the next dry season.

This led Pringle and colleagues to hypothesize that when *C. alliodora* trees get less rain, they incur the cost of supplying more carbon to scale insects but recoup their investment in the currency of better leaf protection by ants. To test their hypothesis, the researchers compared trees at 26 sites from Mexico to Costa Rica where rainfall varied four-fold. As expected, trees at drier sites had both more scales and bigger ant colonies. Amazingly, ants in drier areas also mounted stronger defenses of their trees, finding and chasing away herbivores more vigorously than ants in wetter areas. In keeping with this observation, excluding ants increased leaf herbivory at drier sites but made no difference at a wetter site.

Next, the researchers assessed whether trees in drier areas had less carbon near the end of the dry season. Analysis of carbohydrates stored in stems confirmed that carbon pools were smaller in trees at drier sites than at wetter sites. Further, trees at drier sites showed early signs of carbon stress: more starch had been converted to the sucrose that helps maintain plant turgor during water stress.

When are the carbon costs of ant defense worth it to carbon-stressed *C. alliodora*? To find out, the researchers modeled carbon trading among the players in this mutualistic system under rainy seasons of varying lengths. Comparison of two herbivory models—chronic but low level versus rare but catastrophic—showed that the latter “insurance” model fit the researchers' observations.

The insurance model correctly predicted that shorter rainy seasons reduce trees' carbon pools while increasing their carbon investment in ants. Likewise, ants invest more in leaf protection when rain is scanty, enlarging colonies and allocating more carbon to producing the workers that defend against herbivores. Bolstering the case for the catastrophic insurance model, it also fits the real world. Over the course of six years of field work in the study area, the researchers say most *C. alliodora* trees were fine, but in a few cases all the leaves had been eaten down to the nubs. And, as they point out, even a rare event could pose a real risk to long-lived trees like *C. alliodora*.

Tying their findings together, the researchers propose that mutualism in this system is driven by the combination of an environmental stress (low rainfall) and a biological stress (the risk of catastrophic herbivory), both of which affect carbon futures in plants. They further suggest that the changes in ant behavior at drier sites may reflect genetic adaptation to local conditions. Previous work has divided *A. pittieri* into northern and southern lineages that occupy different precipitation niches, raising the question of whether the rainfall-related differences in ant defensive behaviors revealed in this study also track these two lineages.

Besides showing that water scarcity strengthens mutualism among *C. alliodora*, scale insects, and ants, this work suggests that the adage “hard times make us closer” may apply broadly to mutualisms between plants and animals, with carbon as the common currency. According to the carbon trading model in this study, trees' bet-hedging against irregular rainy seasons may drive the evolution of variable carbon investments.

**Pringle EG, Akçay E, Raab TK, Dirzo R, Gordon DM (2013) Water Stress Strengthens Mutualism Among Ants, Trees, and Scale Insects.** doi:10.1371/journal.pbio.1001705

